# PRP-19, a conserved pre-mRNA processing factor and E3 ubiquitin ligase, inhibits the nuclear accumulation of GLP-1/Notch intracellular domain

**DOI:** 10.1242/bio.034066

**Published:** 2018-07-15

**Authors:** Silvia Gutnik, Yann Thomas, Yanwu Guo, Janosch Stoecklin, Anca Neagu, Lionel Pintard, Jorge Merlet, Rafal Ciosk

**Affiliations:** 1 Friedrich Miescher Institute for Biomedical Research, Maulbeerstrasse 66, 4058 Basel, Switzerland; 2 University of Basel, Petersplatz 1, 4001 Basel, Switzerland; 3 Cell Cycle and Development, Institut Jacques Monod, UMR7592 CNRS - Université Paris Diderot, Sorbonne Paris Cité, F-75013 Paris, France; 4 Department of Biosciences, Faculty of Mathematics and Natural Sciences, University of Oslo, 0316 Oslo, Norway; 5 Sorbonne Université, CNRS, Institut de Biologie Paris-Seine (IBPS), Developmental Biology Laboratory, UMR 7622, F-75005 Paris, France; 6 Institute of Bioorganic Chemistry, Polish Academy of Sciences, Noskowskiego 12/14, 61-704 Poznan, Poland

**Keywords:** *Caenorhabditis elegans*, GLP-1, Notch, PRP-19, Splicing, Ubiquitin ligase

## Abstract

The Notch signalling pathway is a conserved and widespread signalling paradigm, and its misregulation has been implicated in numerous disorders, including cancer. The output of Notch signalling depends on the nuclear accumulation of the Notch receptor intracellular domain (ICD). Using the *Caenorhabditis elegans* germline, where GLP-1/Notch-mediated signalling is essential for maintaining stem cells, we monitored GLP-1 *in vivo*. We found that the nuclear enrichment of GLP-1 ICD is dynamic: while the ICD is enriched in germ cell nuclei during larval development, it is depleted from the nuclei in adult germlines. We found that this pattern depends on the ubiquitin proteolytic system and the splicing machinery and, identified the splicing factor PRP-19 as a candidate E3 ubiquitin ligase required for the nuclear depletion of GLP-1 ICD.

## Abstract (precis)

**Summary:** GFP tagging of endogenous GLP-1/Notch revealed life stage-specific differences in the nuclear accumulation of GLP-1 intracellular domain, which are mediated by the ubiquitin-proteolytic system and the splicing factor/E3 ubiquitin ligase PRP-19.

## INTRODUCTION

Notch signalling is a highly conserved communication pathway with numerous cellular and developmental roles. Consequently, defects in Notch signalling can lead to diverse diseases, including cancer (Artavanis-Tsakonas et al., 1999; Bray, 2006). The signalling depends on the interaction between a Notch receptor and its ligand, which are expressed in neighbouring cells. Upon the binding of a DSL (Delta Serrate LAG-2 family) ligand, the Notch receptor is subjected to sequential cleavages, releasing the intracellular domain (ICD) from the cell membrane. Subsequently, the ICD translocates into the nucleus, where it associates with transcriptional co-activators, regulating transcription of cell type-specific target genes (Bray, 2006).

The strength of Notch signalling must be tightly regulated, as inappropriate dosage of signalling can lead to developmental defects and disease (Andersson and Lendahl, 2014; Berry et al., 1997; Ferrando, 2009; Fiúza and Arias, 2007; Pepper et al., 2003). The mechanisms controlling the output of Notch signalling include modifications of Notch ligands or receptors with ubiquitin, which impact their sub-cellular localization (via selective trafficking) or abundance (via ubiquitin-mediated proteasomal degradation) (Bray, 2006; Pickart and Fushman, 2004).

The ubiquitin-proteolytic system (UPS) requires the coordinated action of three enzymes: E1 ubiquitin-activating and E2 ubiquitin-conjugating enzymes, and E3 ubiquitin ligases. Their activities promote covalent addition of ubiquitin chains to lysine residues of protein substrates, targeting them for subsequent degradation by the 26S proteasome, a large macromolecular complex with protease activities (Hershko et al., 1983). Also, the Notch receptor can be targeted for degradation by the UPS, its specificity depending on a multi subunit E3 ubiquitin ligase, nucleated around the scaffold protein Cullin-1, and using the F-box protein SEL-10/FBXW7 (hereafter SCF^SEL-10^) as the substrate recognition subunit (Killian et al., 2008) [for review on SCF complexes see Cardozo and Pagano (2004)].

There are two *Caenorhabditis elegans* Notch-like receptors: LIN-12 and GLP-1 (Greenwald, 2005; Priess, 2005). Here, we focus on GLP-1, which is essential for the self-renewal of germline stem cells (Austin and Kimble, 1987). In this model, the ligand LAG-2 is provided by the so-called distal tip cell (DTC), which functions as a stem cell niche for the germline (Hubbard, 2007). The receptor, GLP-1, is expressed by a pool of germ cells adjacent to the DTC in the distal-most part of the germline (Crittenden et al., 1994; Henderson et al., 1994). Balancing the dosage of GLP-1 signalling in the germline is important, as too little results in germ cell loss and, conversely, too much leads to tumorous proliferation (Berry et al., 1997; Francis et al., 1995; Kimble and Simpson, 1997; Kodoyianni et al., 1992). In this model, the posttranscriptional regulation of *glp-1* mRNA received most attention (Farley and Ryder, 2012; Kaymak and Ryder, 2013; Kershner and Kimble, 2010; Marin and Evans, 2003; Millonigg et al., 2014; Scheckel et al., 2012; Wright et al., 2011). By contrast, the possible turnover of GLP-1 ICD has remained speculative: while the E3 ligase SCF^SEL-10^ was reported as part of LIN-12 and GLP-1 signalling in embryos, several lines of evidence suggested that it does not play a role in GLP-1 signalling in the self-renewal of germline stem cells (Hubbard et al., 1997; Pepper et al., 2003; Safdar et al., 2016; Sundaram and Greenwald, 1993).

Although ICD is widely assumed to be critical for the germline function of GLP-1, the nuclear accumulation of GLP-1 has not been reported. Here, to visualize it, we GFP-tagged the ICD by CRISPR-mediated genome editing of the endogenous *glp-1* gene. While, as expected, we observed the nuclear accumulation of GLP-1 in germ cells during most larval development, the nuclear GLP-1 was, strikingly, absent from the adult germline. We provide evidence that this nuclear depletion of GLP-1 depends on UPS and uncover PRP-19 as the potential E3 ligase promoting the degradation of GLP-1 ICD. However, PRP-19 has a known function in splicing and the nuclear depletion of GLP-1 also depends on the splicing apparatus. Thus, whether the observed effect of PRP-19 reflects a direct ubiquitination of GLP-1, or is indirectly related to its role in splicing, will need to be determined by future experiments.

## RESULTS

### The nuclear localization of GLP-1 in germ cells is dynamic during development

**Fig. 1.**
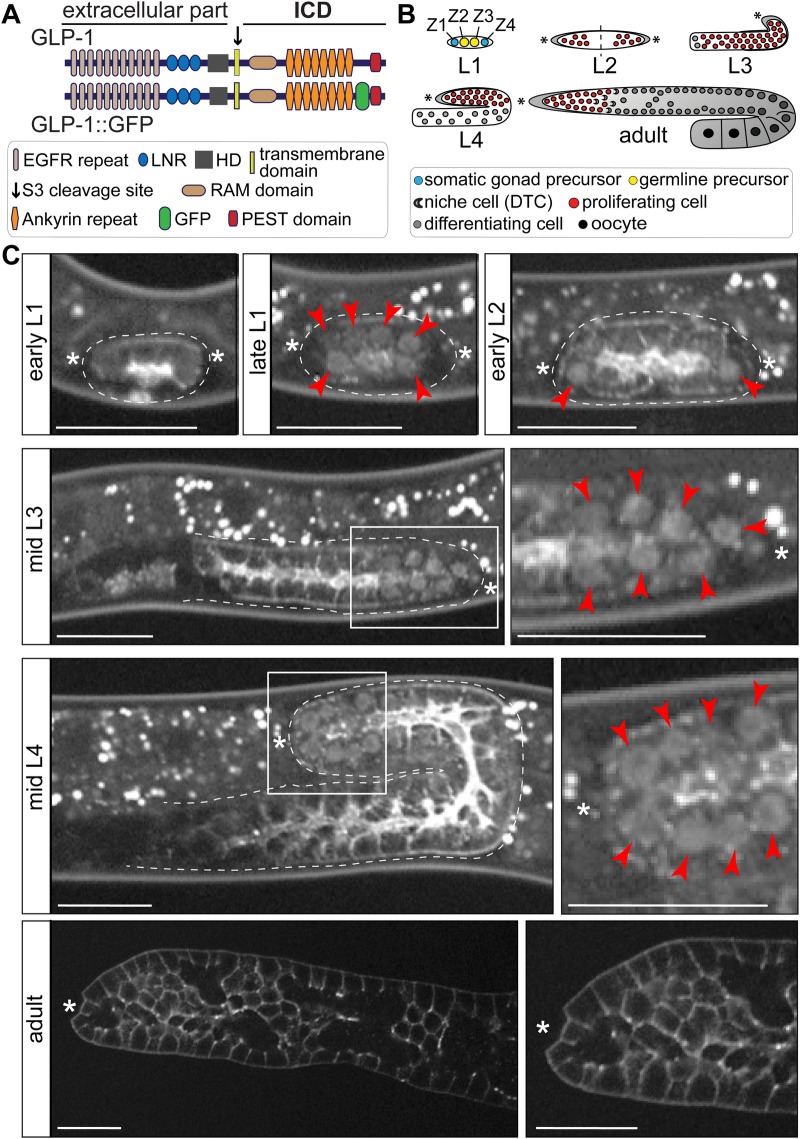
**Nuclear localization of GLP-1 changes during germline development.** (A) Schematic representation of the domain organization of GLP-1 and GLP-1::GFP. The GFP was inserted between the ankyrin-repeats and the PEST domain (*glp-1* allele *rrr27*), within the GLP-1 intracellular domain (ICD). (B) Schematic summarizing the development of *C. elegans* germline. L1–4 indicate stages of larval development, and Z1–4 indicate precursors of the somatic gonad or germline, as indicated. (C) Representative confocal images of worms/gonads expressing GLP-1::GFP, imaged at the indicated developmental stages, from larvae (stages L1–4) to adult. At least 20 animals/gonads were examined per condition. The germlines are outlined with white dotted lines and asterisks mark the approximate locations of distal tip cells (DTCs). Boxed areas (mid L3 and mid L4) are magnified on the right. Adult germlines were dissected before imaging. Images were adjusted with a gamma of 2. Nuclear GLP-1::GFP (indicated by red arrowheads) is detected in proliferating larval germ cells, starting from mid L1 through L4, but not in the adult germ cells, where GFP highlights cell membranes. Scale bars: 20 µm.

Although the essential function of GLP-1 signalling in promoting the self-renewal of germ cells is well established [reviewed in Kimble and Crittenden (2005, 2007)], the nuclear localization of GLP-1 has not been reported. Therefore, to visualize the expected nuclear localization of GLP-1, we generated, by CRISPR-mediated genome editing, a GFP knock-in between the ankyrin repeats and the PEST domain within the GLP-1 ICD [referred to as GLP-1::GFP, allele *glp-1(rrr27)*; Fig. 1A]. The obtained homozygous transgenic animals appeared superficially wild type, as the *glp-1* phenotypes such as sterility, embryonic lethality, or tumorous germlines were not observed. In agreement with the previously published expression pattern of GLP-1 using antibodies (Crittenden et al., 1994), and its function as a cell membrane receptor, we observed the GLP-1::GFP on cell membranes throughout germline development (Fig. 1B–C). Additionally, we observed the nuclear GLP-1::GFP, presumably corresponding to the activated ICD part of the protein, in most larval gonads, with the exception of newly hatched L1 larvae (Fig. 1C). This latter observation is consistent with earlier findings that *glp-1* is dispensable for the first mitotic division of germline precursors in L1 larvae (Austin and Kimble, 1987). Surprisingly, however, despite the essential proliferation-promoting role of GLP-1, the nuclear GLP-1::GFP was not observed in adults (Fig. 1C), suggesting the existence of a mechanism restricting the nuclear accumulation of GLP-1 ICD in adult gonads.

### The UPS prevents the nuclear accumulation of GLP-1

**Fig. 2.**
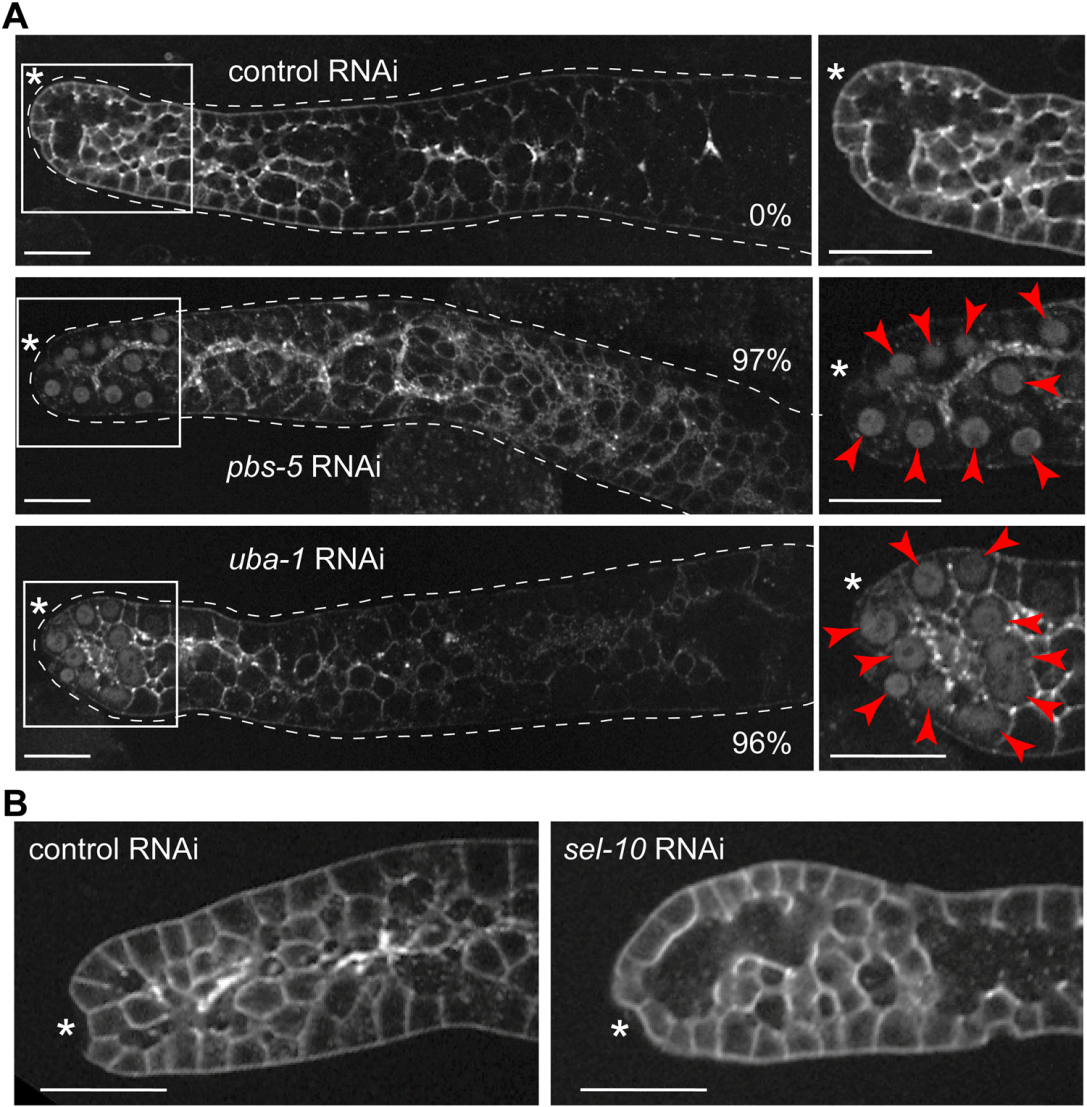
**The nuclear localization of GLP-1 is prevented by the ubiquitin-proteolytic-system (UPS).** (A) Representative confocal images of gonads dissected from *glp-1(rrr27)* adult worms, subjected to either control RNAi, or RNAi against *pbs-5* or *uba-1*. While the control gonads did not display the nuclear GFP (0%; *n*=47), the nuclear GFP was observed in 97% (*n*=30) or 96% (*n*=24) gonads, from *pbs-5(RNAi)* or *uba-1(RNAi)* worms, respectively (red arrowheads). The germlines are outlined with white dotted lines. Boxed areas on the left are magnified on the right. (B) Representative confocal images of gonads dissected from *glp-1(rrr27)* worms, subjected to either control or *sel-10* RNAi. The depletion of *sel-10* did not lead to the nuclear enrichment of GLP-1::GFP (*n*=10 for *sel-10* and *n*=47 for control RNAi). Asterisks mark DTCs. Images were adjusted with a gamma of 2. Scale bars: 20 µm.

The observed deficit of nuclear GLP-1 in adult gonads could be explained by a proteolytic degradation of GLP-1, as was shown for other Notch receptors (Bray, 2006). To test whether the nuclear GLP-1 depends on the UPS, we RNAi-depleted *pbs-5* (a proteasome component) or *uba-1* (the only E1 conjugating enzyme encoded by the *C. elegans* genome). We observed a very high incidence of nuclear GLP-1::GFP in both *pbs-5(RNAi)* and *uba-1(RNAi)* gonads (respectively in 97% and 96% of the gonads), but not the control gonads (Fig. 2A). In UPS, substrate specificity is conferred by distinct E3 ligases. The E3 ligase SCF^SEL-10^ is known to target Notch ICD for degradation in several systems, including worms, but possibly excluding the worm germline stem cells (Hubbard et al., 1997; Safdar et al., 2016; Sundaram and Greenwald, 1993). To confirm this, we RNAi-depleted *sel-10* and found that its depletion did not lead to the nuclear accumulation of GLP-1::GFP (Fig. 2B). Consistently, neither RNAi-depletion of *cul-1* and *rbx-1*, which are part of the SCF^SEL-10^ E3 ligase, lead to the nuclear accumulation of GLP-1::GFP (Fig. S1).

### RNAi screen targeting E3-ligases reveals PRP-19 as a factor important for the nuclear depletion of GLP-1

**Fig. 3.**
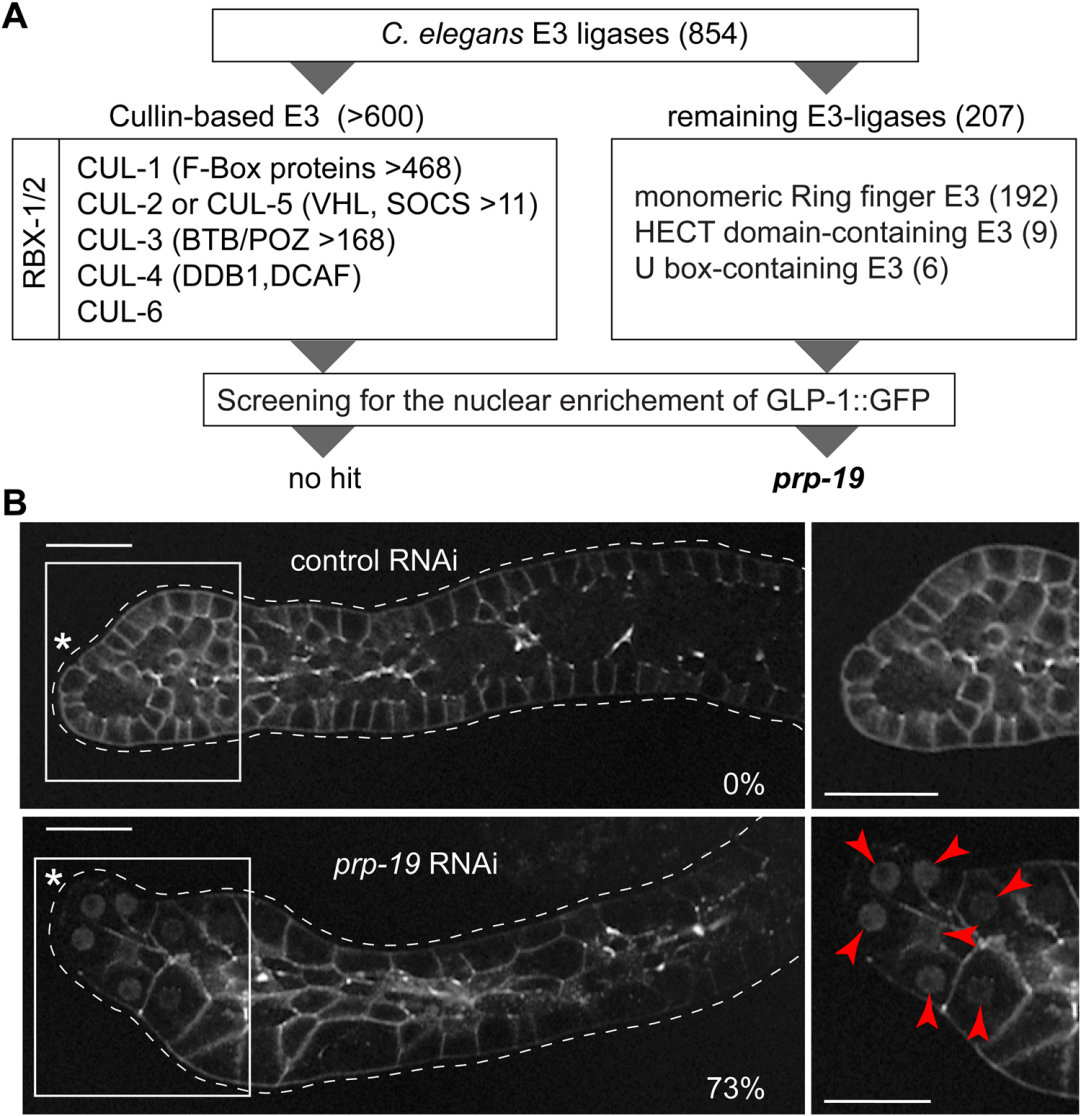
**The nuclear localization of GLP-1 is inhibited by the putative E3-ligase PRP-19.** (A) Schematic representation of the E3 ligase screen performed in this study. The list of E3 ligases encoded by the *C. elegans* genome is derived from (Gupta et al., 2015). Depleting the six *C. elegans* cullin family members, *cul-1* to *cul-6*, as well as *rbx-1* and *rbx-2*, did not result in the nuclear enrichment of GLP-1::GFP, as assessed by fluorescent microscopy on dissected gonads (Fig. S1). Knocking-down other E3-ligases similarly did not lead to the nuclear enrichment of GLP-1::GFP, with the only exception of *prp-19(RNAi)*. (B) Representative confocal images of gonads dissected from adult *glp-1(rrr27)* worms, subjected to either control or *prp-19* RNAi*.* The germlines are outlined with white dotted lines. Boxed areas on the left are magnified on the right and asterisks indicate DTCs. Images were adjusted with a gamma of 2. While the control gonads did not show the nuclear enrichment of GLP-1::GFP (0%; *n*=47), 73% of *prp-19(RNAi)* gonads (*n*=44) showed the nuclear enrichment (red arrowheads). Scale bars: 20 µm.

In order to identify putative E3 ligase(s) preventing the nuclear accumulation of GLP-1, we RNAi-depleted most of the *C. elegans* E3 ligases and searched for the nuclear enrichment of GLP-1::GFP in adult gonads. The *C. elegans* genome encodes 854 putative E3 ligases, as determined by protein domain analysis (Gupta et al., 2015). The cullin-RING E3 ligases (CRLs) are the most prominent class of ubiquitin ligases (Merlet et al., 2009). CRLs are multi-subunit complexes, nucleated around a cullin scaffold protein (CUL-1 to CUL-6) and contain one adaptor protein RBX-1 or RBX-2 (Fig. 3A). Thus, to test the possible involvement of CRLs, we RNAi-depleted *rbx-1* or *rbx-2,* as well as (individually) all six cullin-family members (>30 dissected adult gonads were tested per RNAi). However, none of these facilitated the accumulation of nuclear GLP-1::GFP (Fig. 3A; Fig. S1). Next, we tested 117 of the remaining 207 putative E3-ligases, which were found to be germline-enriched [(Scheckel et al., 2012) and Table S1], and found that depleting PRP-19, a conserved monomeric U-box E3-ligase, resulted in the nuclear enrichment of GLP-1::GFP in most (73%) gonads (Fig. 3B).

### *prp-19* interacts genetically with *glp-1*

**Fig. 4.**
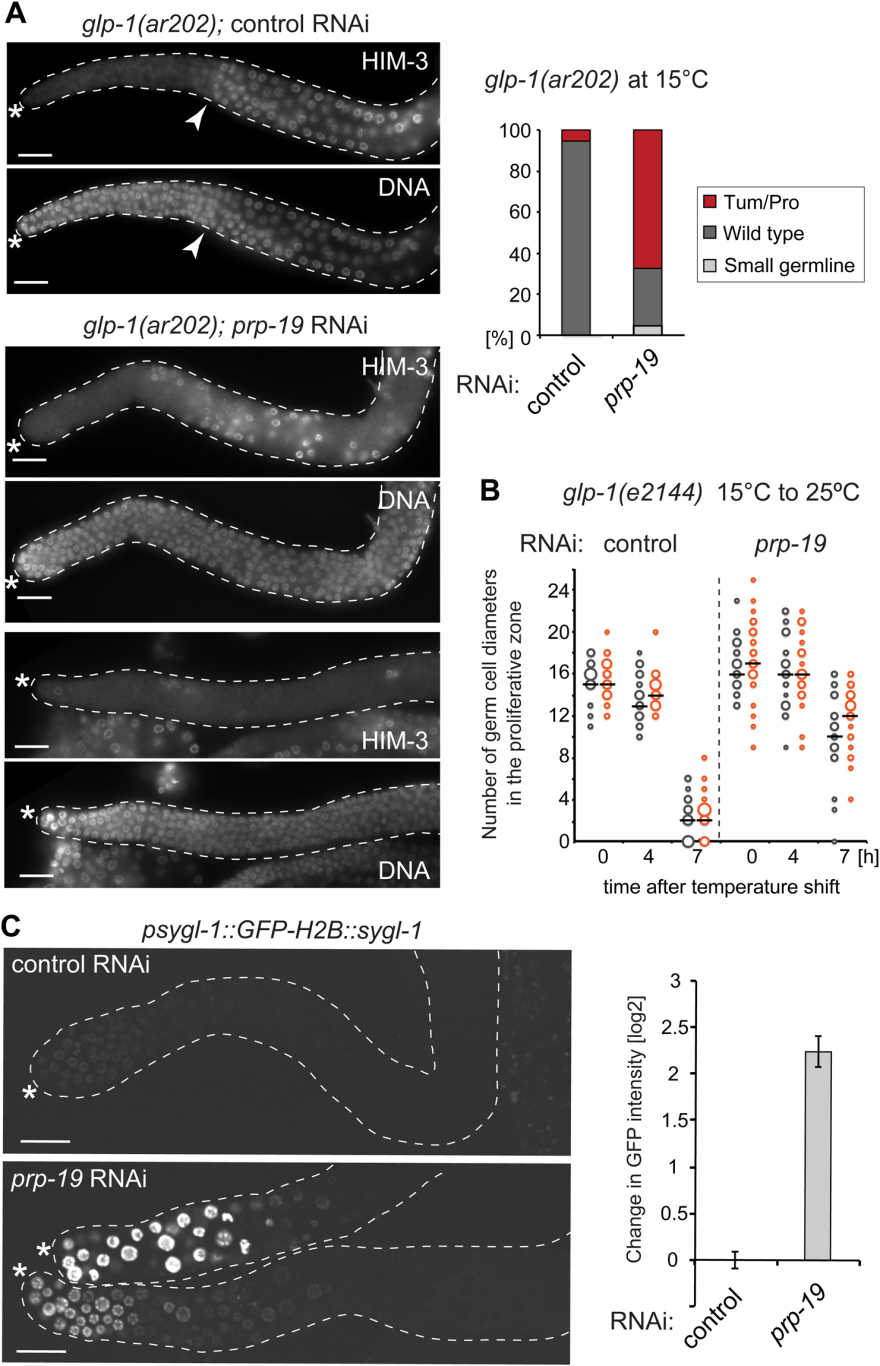
***prp-19* interacts genetically with *glp-1*.** (A) Depletion of *prp-19* enhances germ cell proliferation in the gain-of-function *glp-1(ar202)* animals at the permissive temperature (15°C). Left: Anti-HIM-3 and DNA (DAPI) stained gonads, dissected from *glp-1(ar202)* animals grown at the permissive temperature, and subjected to either control or *prp-19* RNAi. While the control-treated *glp-1(ar202)* gonads were superficially wild type (top panels; white arrowheads mark the transition from mitosis to meiosis), the *prp-19(RNAi)* gonads contained ectopic proliferative cells (HIM-3 negative) away from the DTC; this phenotype ranged from mild (middle panel) to fully penetrant germline tumours (bottom panel). Top right: Quantification of the *glp-1(ar202)* germline phenotypes at 15°C, upon control (*n*=37) or *prp-19* (*n*=43) RNAi. ‘Small germline’ indicates gonads with decreased numbers of germ cells, while ‘Tum/Pro’ (Tumorous/Proximal) indicates gonads with proliferating cells either throughout the gonad or in the proximal gonad, respectively. (B) Depletion of *prp-19* delays the loss of proliferating germ cells in the temperature-sensitive, loss-of-function, *glp-1(e2144)* germline. The size of the proliferative zone [expressed as the number of so-called germ cells diameters; (Crittenden and Kimble, 2008)], was measured from the distal end of the germline to the first row of germ cell nuclei containing at least two crescent shaped nuclei, indicative of entry into meiosis. Shown are the results of two independent experiments (indicated by different colours; *n*>20 per each experimental condition). The black bars indicate averages and the size of the dots is proportional to the number of times that a given length was recorded. Note that *prp-19(RNAi)* germlines retained mitotic cells even after 7 h at 25°C (*P*-value 1.6×10^−11^/1.8×10^−19^ for the two respective experiments at 7 h). (C) Depletion of *prp-19* results in the up-regulation of a GLP-1 target gene, *sygl-1*. On the left are representative confocal images of gonads dissected from worms expressing the *psygl-1::GFP::H2B::sygl-1* reporter, which were subjected to either control or *prp-19* RNAi. The corresponding quantifications are on the right (*n*=23 for *prp-19* and *n*=19 for control RNAi). In all images, the gonads are outlined with white dotted lines and asterisks mark DTCs. Scale bars: 20 µm.

Genetic interactions between mutant alleles of different genes can indicate functional relationship. While the loss of GLP-1 function leads to a loss of mitotic germ cells, its hyper-activation results in germ cell over proliferation and, consequently, germline tumours (Austin and Kimble, 1987; Hansen and Schedl, 2006; Hansen et al., 2004). To examine the potential functional relationship between PRP-19 and GLP-1, we examined the effect of PRP-19 depletion on germ cell proliferation in animals carrying conditional gain- or loss-of-function alleles of *glp-1*. Firstly, we examined the effect of PRP-19 depletion on animals carrying a temperature-sensitive, gain-of-function allele of *glp-1*, *glp-1*(*ar202*) (Pepper et al., 2003). At the permissive temperature (15°C) only 5% of *glp-1(ar202)* germlines developed tumours. However, when combined with *prp-19(RNAi)*, the number of tumorous gonads increased to 67% (Fig. 4A). Secondly, we examined the gonads from *prp-19(RNAi)* animals carrying a temperature-sensitive loss-of-function *glp-1* allele, *glp-1(e2144)* (Priess et al., 1987)*.* At the permissive temperature (15°C), the germlines of *glp-1(e2144)* animals had a proliferative zone of about 15–16 germ cell diameters (Fig. 4B). When shifted to the restrictive temperature (25°C), the proliferative zone progressively decreased, nearly disappearing by 7 h (Fig. 4B). Impressively, under the same conditions, depleting PRP-19 significantly delayed the loss of proliferative cells in *glp-1(e2144)* animals (Fig. 4B); at 7 h at 25°, the proliferative zone in *glp-1(e2144); prp-19(RNAi)* gonads was, on average, 10 or 12 germ cell diameters, in two independent experiments, respectively (Fig. 4B). Thus, the depletion of PRP-19 partially rescued the loss of *glp-1*, while enhancing *glp-1* overstimulation. Finally, to examine the effect of PRP-19 on GLP-1 in proliferating germ cells more directly, we examined, following PRP-19 depletion, the expression of one of two redundant*,* proliferation-promoting GLP-1 target genes, *sygl-1* (Kershner et al., 2014). To facilitate detection, we used a reporter expressing GFP::H2B (concentrating GFP in the nuclei) from the *sygl-1* promoter and under the control of *sygl-1* 3'UTR (Kershner et al., 2014). Interestingly, we observed a strong upregulation of this reporter following *prp-19* RNAi (Fig. 4C), while an unrelated reporter was not affected (Fig. S2). Combined, these observations suggest that PRP-19 acts as a negative regulator of GLP-1 signalling in the germline, which is potentially consistent with PRP-19-induced proteasomal degradation of GLP-1 ICD.

To examine the localization of PRP-19, we generated, by CRISPR mediated genome editing, a strain expressing Strep-tagged PRP-19 [allele *prp-19(rrr25)*]. Using antibodies against the Strep tag, we observed nuclear localization of PRP-19 throughout the germline (Fig. S3). Prp19 proteins are highly conserved and the key residues required for the ubiquitin ligase activity are either invariant or conserved in the U-box of PRP-19 (Ohi et al., 2003; Vander Kooi et al., 2006) (Fig. S4), suggesting that also PRP-19 may function, in germ cell nuclei, as an E3-ligase.

### Prp19 complex and splicing machinery prevent the nuclear accumulation of GLP-1

**Fig. 5.**
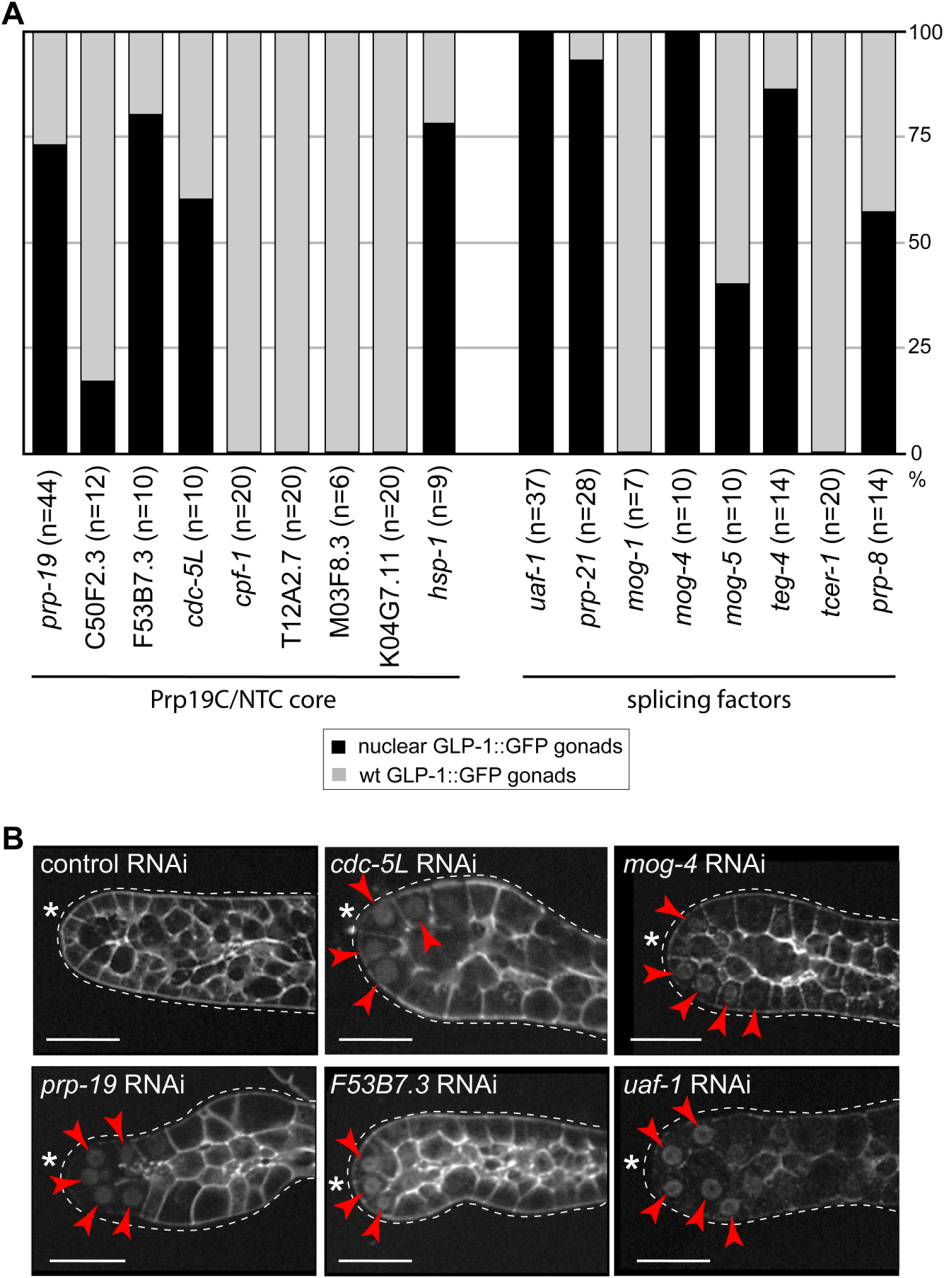
**Depleting components of the *C. elegans* Prp19C/NTC complex, or splicing factors, results in the nuclear enrichment of GLP-1.** (A) Percentage of gonads, from animals of the indicated genotypes, displaying the nuclear GLP-1::GFP. The gonads were dissected from *glp-1(rrr27)* animals and examined for the nuclear GFP, upon RNAi-mediated depletion of putative components of the Prp19C/NTC complex (left) or unrelated splicing factors (right). ‘n’ indicates the numbers of examined gonads. (B) Representative examples of confocal images from A. Red arrowheads point to the nuclear GFP. The gonads are outlined with white dotted lines and asterisks (*) mark DTCs. Images were adjusted with a gamma of 2. Scale bars: 20 µm.

Prp19 is central to a large protein complex, known as the Prp19 complex or NineTeen Complex (Prp19C/NTC). Prp19C/NTC consist of eight core proteins and up to 19 associated proteins in yeast, and more than 30 proteins in higher eukaryotes, including in animals and plants [for review see (Chanarat and Sträßer, 2013) and (Ambrósio et al., 2015); Table S2]. Although this complex remains uncharacterized in *C. elegans*, we RNAi-depleted several putative worm components of the Prp19C/NTC to test whether the PRP-19 effect on nuclear GLP-1::GFP may involve Prp19C/NTC. We found that the depletion of several putative Prp19C/NTC components promoted the nuclear accumulation of GLP-1::GFP, with varying penetrance (Fig. 5); at the same time, their depletion did not alter the overall abundance or nuclear localization of PRP-19::STREP (data not shown). The best-characterized role of Prp19C/NTC is in splicing. Thus, we additionally investigated GLP-1::GFP localization following RNAi-mediated depletion of several splicing factors functioning outside the Prp19C/NTC complex. We observed that depleting several such splicing factors lead to the nuclear enrichment of GLP-1::GFP, with varying penetrance (Fig. 5), suggesting that PRP-19-mediated control of nuclear GLP-1 may reflect its function in splicing.

## DISCUSSION

### Nuclear localization of GLP-1 in the germline

The germline GLP-1 was previously shown, by indirect immunofluorescence, to localize to cell membranes, even when using antibodies raised against the ankyrin repeats within ICD (Crittenden et al., 1994). Nevertheless, SEL-12 or HOP-1 presenilins, the catalytic subunits of the gamma secretase that cleaves GLP-1, are required for the germline function of GLP-1 (Francis et al., 2002; Li and Greenwald, 1997; Westlund et al., 1999), suggesting that releasing GLP-1 ICD from the cell membrane takes place also in this tissue. Our tagging strategy potentially allowed visualization of the GLP-1 ICD (in full-length or cleaved protein), avoiding problems potentially associated with using antibodies. The observed nuclear enrichment of GLP-1::GFP in a few distal-most nuclei is consistent with a recent report suggesting that GLP-1 targets are most likely to be activated in the most distal germ cells (Lee et al., 2016). However, although the nuclear signal is highly suggestive, we have no direct proof that the nuclear GLP-1::GFP corresponds to the activated GLP-1 ICD.

### Life stage-specific nuclear localization of GLP-1

While we observed the anticipated nuclear localization of GLP-1 ICD during most of germline development, we noticed life stage-specific exceptions, seemingly inconsistent with the postulated absolute requirement for GLP-1 ICD in maintaining germ cell proliferation. However, the absence of nuclear GLP-1 in the youngest L1s is consistent with a delayed onset of germ cell proliferation in these larvae, and the fact that GLP-1 is dispensable for the first round of germline proliferation (Austin and Kimble, 1987). The absence of nuclear GLP-1 ICD in adult germlines is more difficult to explain. In one scenario, the nuclear GLP-1 would be dispensable for germ cell proliferation in adults. However, agreeing with the generally accepted model of GLP-1/Notch signalling, the processing of GLP-1 by HOP-1/presenilin, expectedly facilitating the release of ICD, takes place also in the adult germline (Agarwal et al., 2018). Thus, we suspect that, while the GLP-1 ICD is essential, its levels are below the detection limits of live imaging. In this scenario, the higher levels of GLP-1 ICD in larvae could promote a more robust proliferation driving germline expansion. Somewhat consistent with this hypothesis, the larval function of GLP-1 requires, in addition to HOP-1, another presenilin, SEL-12 (Agarwal et al., 2018), possibly enhancing the production of GLP-1 ICD.

### Splicing and the nuclear levels of GLP-1

Our analysis suggests that the splicing machinery, directly or indirectly, is involved in the regulation of nuclear GLP-1. In fact, splicing factors have been implicated in the regulation of germline proliferation (Kerins et al., 2010; Mantina et al., 2009; Wang et al., 2012), and they were proposed to function downstream from, or parallel to, GLP-1 (Kerins et al., 2010; Mantina et al., 2009). However, the relevant miss-spliced mRNAs have not been identified so far (Belfiore et al., 2004; Puoti and Kimble, 2000). One possibility might be that the *glp-1* mRNA is itself subjected to alternative splicing, and that the nuclear GLP-1 might reflect a splice variant, rather than the product of protein processing. However, a shorter protein corresponding to the ICD, produced independently of the canonical protein processing, would be expected to induce a constitutive Notch signalling, expectedly leading to germline tumours. Also, the germline function of GLP-1 requires both the activation by DSL ligand(s), and the cleavage by gamma secretases in larvae and adults (Agarwal et al., 2018; Francis et al., 2002; Li and Greenwald, 1997; Westlund et al., 1999). Moreover, specific mutations in the GLP-1 transmembrane domain (oz25, q158 and q172) result in *glp-1* null-like phenotypes (Austin and Kimble, 1987; Kodoyianni et al., 1992). Finally, we find no evidence for alternatively spliced *glp-1* isoforms based on published developmental time-course RNAseq experiments [(Hendriks et al., 2014); Fig. S5]. Therefore, the existence of a short GLP-1 isoform made by an alternative splicing is unlikely. Instead, efficient splicing may be required for the production of factor(s) limiting the nuclear accumulation of GLP-1. However other explanations are possible, as discussed below.

### Potential ubiquitination of GLP-1 ICD by PRP-19

Although the E3 activity of PRP-19 remains to be demonstrated, the homologous proteins are known to function as E3s, and key residues required for the E3 activity are conserved in PRP-19 (Ohi et al., 2003; Vander Kooi et al., 2006). Testing the possible E3 role of PRP-19, we expressed it alongside GLP-1 ICD in mammalian cells but observed no ubiquitination (data not shown). Nonetheless, Prp19 has been recently reported to require components of the Prp19C/NTC complex for the E3 activity (de Moura et al., 2018). Thus, it is likely that also PRP-19 requires co-factors to function as E3. Along these lines, depleting the worm counterparts of Prp19C/NTC components (as in Fig. 5) could compromise the E3 activity of PRP-19. Moreover, Prp19C/NTC is essential for the catalytic activation of the spliceosome [review in (Chanarat and Sträßer, 2013)]. All things considered, separating the putative E3 activity of PRP-19 from Prp19C/NTC and splicing may be very difficult. Thus, although our results imply the regulation of nuclear GLP-1 by splicing, it remains possible that PRP-19 might regulate GLP-1 independently from its role in splicing. In one particularly interesting potential scenario, PRP-19, while engaged in splicing, could directly promote the ubiquitination and degradation of promoter-associated GLP-1 ICD, possibly providing a negative feedback mechanism controlling the transcriptional output of GLP-1 ICD. At this stage, however, this and other models remain speculative, and future experiments are required to explain how exactly PRP-19 regulates the nuclear abundance of GLP-1.

## MATERIALS AND METHODS

### Nematode culture and mutants

Standard procedures were used to maintain animals (Brenner, 1974). Worms were grown at 20°C unless stated otherwise. All temperature-sensitive strains were kept at 15°C. Strains with the following genotypes were used: N2 bristol (wild type); *glp-1(e2144)*III (Priess et al., 1987); *glp-1(ar202)*III (Pepper et al., 2003); qSi26[*psygl-1::GFP::H2B::sygl-1 unc-119(+)*] II; unc-119(ed3) III; teIs1 IV (Kershner et al., 2014)*;* weSi6[Pmex-5::H2B::GFP unc-119(+)](Zeiser et al., 2011); *glp-1(rrr27[glp-1::gfp])*III (this study); *prp-19(rrr25[prp-19::strep])*III (this study).

### CRISPR mediated genome editing

Genome editing at the *glp-1* locus was performed as described previously (Dickinson et al., 2013; Katic and Großhans, 2013), and genome editing at the *prp-19* locus was done as described elsewhere (Arribere et al., 2014). sgRNA used for Crispr/Cas9: SG176_sgRNA_Notch ICD_V3_F (5′-AAT TGC AAA TCT AAA TGT TTG TGA AGA ATA TCA AAA GAG CGT TTT AGA GCT AGA AAT AGC-3′); SG177_sgRNA_Notch ICD_V3_R (5′- GCT ATT TCT AGC TCT AAA ACG CTC TTT TGA TAT TCT TCA CAA ACA TTT AGA TTT GCA ATT-3′); SG474_sgRNAi_prp19_F1 (5′- AAT TGC AAA TCT AAA TGT TTgt gta tat ttt gct act ttc GTT TAA GAG CTA TGC TGG AA-3′); SG475_sgRNAi_prp19_R1 (5′-TTC CAG CAT AGC TCT TAA ACg aaa gta gca aaa tat aca cAA ACA TTT AGA TTT GCA ATT-3′); SG476_sgRNAi_prp19_F2 (5′-AAT TGC AAA TCT AAA TGT TTa caa TTA GAA AGA GAA TAC TGT TTA AGA GCT ATG CTG GAA-3′); SG477_sgRNAi_prp19_R2 (5′- TTC CAG CAT AGC TCT TAA ACA GTA TTC TCT TTC TAA ttg tAA ACA TTT AGA TTT GCA ATT-3′).

### RNAi interference experiments

RNAi mediated knock-down was performed by feeding the animals with bacteria containing RNAi clones from the Ahringer and Vidal (OBS) RNAi libraries as stated in Table S1 (Kamath et al., 2001; Reboul et al., 2003). Experiments were performed at 25°C using overnight-synchronized L1 animals or staged L4 animals as stated in Table S1. For control, RNAi bacteria containing empty feeding vector L4440 were used. For temperature shift experiments, *glp-1(e2144)* worms, synchronized as L1s, were cultured to young adult stage on RNAi inducing plates at 15°C, before they were shifted to 25°C for the indicated time.

### E3 ligase screen

The list of RNAi clones for the E3 ligases screened is stated in Table S1. Screening for nuclear enrichment of GLP-1::GFP in the germline was done with a Zeiss Axio Imager Z1 microscope. Confocal imaging was used for illustrations.

### Confocal imaging

Confocal images were captured with Axio Imager M2 (upright microscope) and the Yokogawa CSU W1 Duel camera T2 spinning disk confocal scanning unit. Images subject to direct comparison were taken at identical exposure times and were processed with Adobe Photoshop CS5.1 in an identical manner. To enhance the contrast for a better visualization of the images in Figs 1, 2, 3, 5 and Fig. S1, we adjusted them with a gamma of 2.

### Analysis of glp-1 mutant germlines

For *glp-1(e2144)*, the size of the proliferative zone [expressed as the number of so-called germ cell diameters, gcd, (Crittenden and Kimble, 2008)], corresponds to the distance between the distal end of the germline, and the appearance of crescent-shaped nuclei (visualized by DAPI) indicative of entry into meiosis. In *glp-1(ar202)* germlines we evaluated, at 15°C, the appearance of non-meiotic cells in the meiotic region of the germline (Turn/Pro germlines). Meiotic germ cells were visualized by HIM-3 immunostaining (a meiotic marker).

### Immunostaining and antibodies

Microscopic slides (three-well diagnostic slides, Thermo Fisher Scientific) were covered with subbing solution containing gelatin (0.4 g/100 ml), chrome alum (0.4 g/100 ml) and Poly-L-Lysine (1 mg/ml, Sigma-Aldrich), and dried for a couple of hours. Worms were dissected with a syringe in a drop of 50% M9 in ddH2O containing Levamisol (300 µM final concentration) and staining was performed as previously described (Burger et al., 2013) with the exception of using Tween-20 instead of Triton-X-100. Working dilutions for the primary antibodies were 1:500 for rabbit anti-HIM-3 (Goodyer et al., 2008) and 1:2000 Strep MAB classic Chromeo 546 (IBA Lifesciences, Goettingen, Germany). For anti-HIM-3 stainings, slides were later incubated for 30 min at RT with secondary antibodies goat anti-rabbit (IgG) coupled to the Alexa 568 fluorophore (1:500, Invitrogen). Next, germlines were mounted in Vectashield Mounting Medium with DAPI (Vector Laboratories, Peterborough, UK).

### Reporter GFP quantifications

Reporter GFP quantifications were done as described (Seelk et al., 2016). Briefly, fluorescent micrographs were recorded with Zeiss Axio Imager Z1 microscope and a Zeiss Axiocam MRm REV 2 CCD camera was used to capture images. For each germline (*n*=19 in control RNAi, *n*=23 in *prp-19(RNAi)* and *n*=19 in Fig. S2), three nuclei from the distal-most zone were taken and intensities quantified using Fiji. Then GFP intensities were normalized to the picture background and corrected with the average autofluorescence measured in wild-type (N2) gonads at the corresponding temperatures. Images subject to direct comparison were taken at identical exposure times and were processed with Adobe Photoshop CS5.1 in an identical manner.

### Ratios of 5′ and 3′ exons of glp-1 during development

The RNA-seq data for *C. elegans* time course development from Hendricks et al. (Hendriks et al., 2014) was used to analyse possible transcript variants of *glp-1*. The processing of the RNA-seq data was as described (Hendriks et al., 2014). The raw read coverage of each exon of *glp-1* was counted with BEDtools (Quinlan and Hall, 2010). The read count ratios are the raw read counts of the first 3 exons of *glp-1*, divided by the raw read counts from other exons.

### Statistical analysis

All *P*-values were calculated using a Student's *t*-test. All calculations were performed in Excel (Microsoft).

## Supplementary Material

Supplementary information
