# Supplementary material for: PRP-19, a conserved pre-mRNA processing factor and E3 ubiquitin ligase, inhibits the nuclear accumulation of GLP-1/Notch intracellular domain
Source: Biol Open. 2018 Jul 15;7(7):bio034066. doi: 10.1242/bio.034066 (PMC6078339; doi:10.1242/bio.034066)
Supplement: Supplementary information [file biolopen-7-034066-s1.pdf]

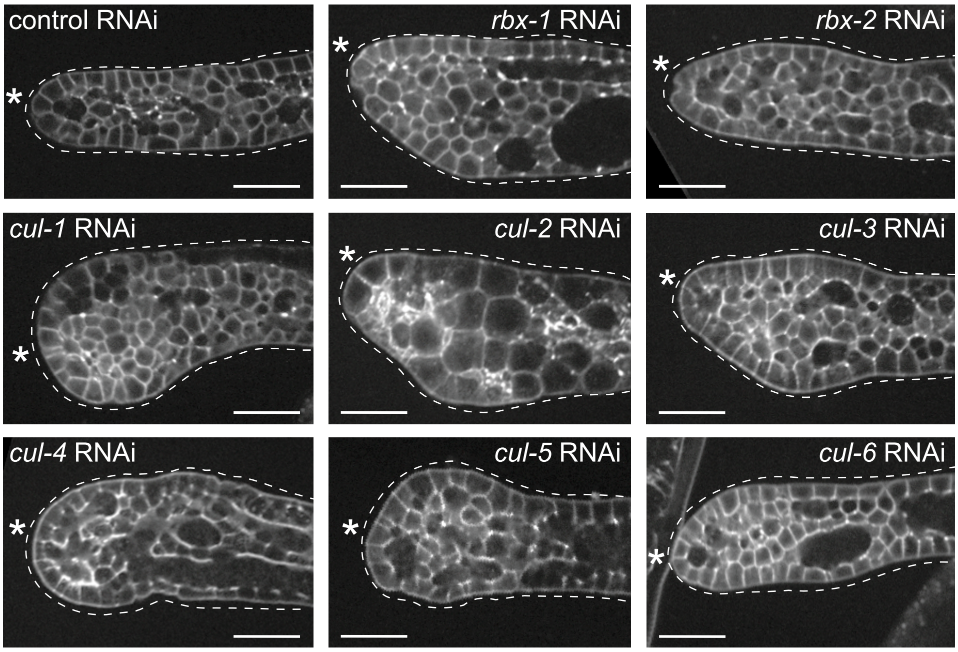

Figure S1: Depletion of Cullin-RING based E3-ligases does not result in the nuclear accumulation of GLP-1

Representative confocal images of gonads dissected from *glp-1(rrr27)* animals, subjected to RNAi as indicated (n>30 per RNAi). Gonads are outlined with white dotted lines and "*" mark DTCs. Images were adjusted with a gamma of 2. Scale bars represent 20 µm. Depletion of the tested factors did not result in the nuclear accumulation of GLP-1::GFP.

Biology Open • Supplementary information

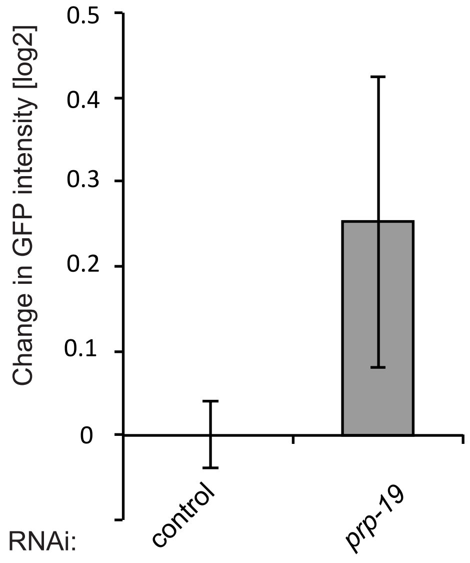

Figure S2: Depletion of *prp-19* does not result in the upregulation of a *pmex-5::GFP::H2B::tbb-2* reporter

Shown are GFP quantifications, from confocal images, of gonads dissected from worms expressing the *pmex-5::GFP::H2B::tbb-2* reporter, which were subjected to either control or *prp-19* RNAi (n=19 for both). By contrast to the *sygl-1* reporter (Fig. 4C), this reporter was not affected by depleting *prp-19* (p-value 0.0109).

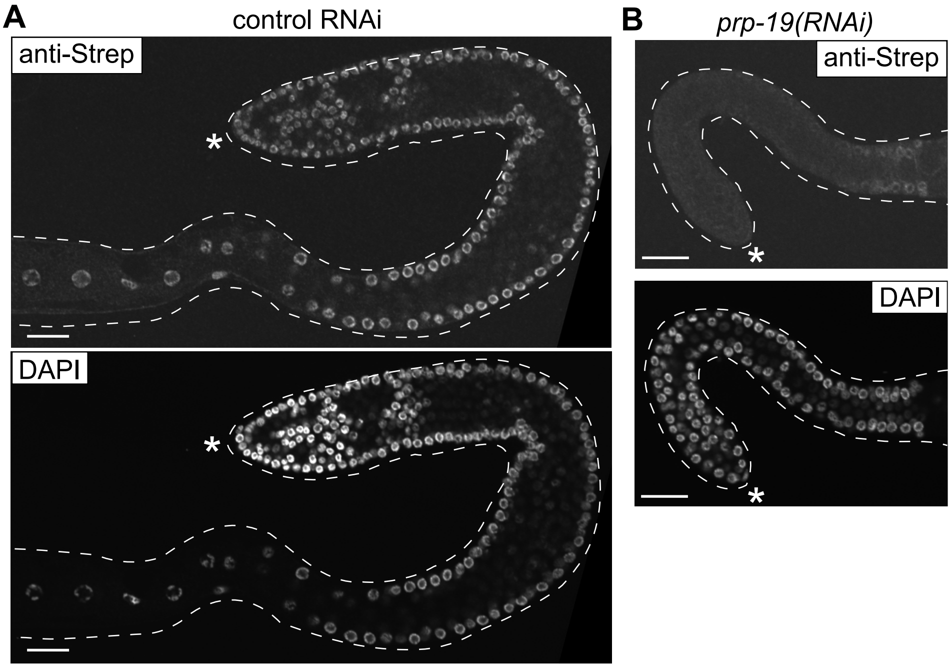

Figure S3: PRP-19 is expressed in germ cell nuclei
Representative confocal images of gonad dissected from adult *prp-19(rrr25)* worms stained for the Strep tag (anti-Strep) and DNA (DAPI), subjected to either control in A or *prp-19* RNAi in B. While PRP-19::Strep localized to the germ cell nuclei, the staining was lost upon *prp-19* RNAi, indicating staining specificity. The gonads are outlined with white dotted lines and “*” mark DTCs. Scale bars represent 20 µm.

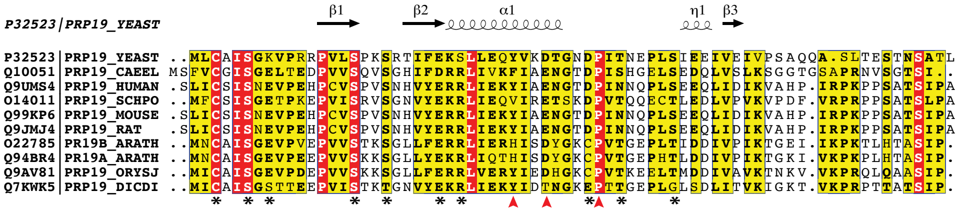

Figure S4: Sequences alignments of PRP19 U-box domains

The alignments were prepared using the uniprot server (The UniProt Consortium, 2017). The resulting alignment file was then processed through ESPript (Robert and Gouet, 2014). Highly similar residues are in red, similar residues are black bold and boxed in yellow. The residues structurally participating in the two hydrogen-bonding network and in the putative E2 interface are marked, respectively, with “*” and red arrowheads.

Biology Open • Supplementary information

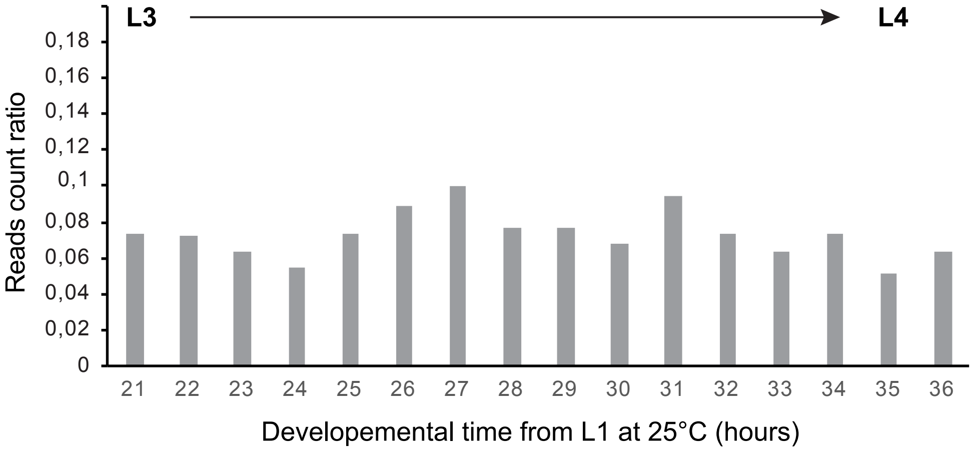

Figure S5: Ratios of 5' and 3' exons of *glp-1* during development
Shown are ratios of the first three *glp-1* exons to the remaining exons, calculated from the developmental time-course RNAseq experiments from Hendricks et al. (Hendriks et al., 2014). Note that there is little or no change in the ratio, arguing against putative 5' isoforms of *glp-1*.

Table S1. List of RNAi clones used in this study

Sequence Name	Gene Name	Remark	RNAi Source	L1	L4
	pbs-5	ubiquitin proteolytic system	OBS		x
	uba-1	ubiquitin proteolytic system	Ahringer library		x
	cul-1	Cullin complex member	Ahringer & OBS	x	x
	cul-2	Cullin complex member	Ahringer & OBS	x	x
	cul-3	Cullin complex member	Lionel Pintard	x	
	cul-4	Cullin complex member	Ahringer library	x	
	cul-5	Cullin complex member	Ahringer & OBS	x	
	cul-6	Cullin complex member	Ahringer & OBS	x	
	rbx-1	Cullin complex member	Ahringer library	x	x
	rbx-2	Cullin complex member	Ahringer library	x	
	sel-10	Cullin complex member	Ahringer library	x	
C50F2.3		Prp19C/NTC	OBS		x
F53B7.3		Prp19C/NTC	Ahringer library	x	
	cdc-5L	Prp19C/NTC	OBS		x
	cpf-1	Prp19C/NTC	Ahringer library	x	
T12A2.7		Prp19C/NTC	Ahringer library	x	
M03F8.3		Prp19C/NTC	Ahringer library		x
K04G7.11		Prp19C/NTC	OBS	x	
	hsp-1	Prp19C/NTC	Ahringer & OBS		x
	uaf-1	Splicing factors	OBS		x
	prp-21	Splicing factors	OBS		x
	mog-1	Splicing factors	Ahringer library	x	
	mog-4	Splicing factors	OBS		x
	mog-5	Splicing factors	Ahringer library		x
	teg-4	Splicing factors	Ahringer library		x
	tcer-1	Splicing factors	Ahringer library	x	
	prp-8	Splicing factors	Ahringer library		x
**E3 ligases screened**					
B0281.3	.	RING-finger	Ahringer library	x	
B0393.6	.	RING-finger	Ahringer library	x	
B0416.4	.	RING-finger	Ahringer & OBS	x	
C01G6.4	.	RING-finger	Ahringer & OBS	x	
C02B8.6	.	RING-finger	OBS	x	
C06A5.8	.	RING-finger	OBS	x	
C06A5.9	*rnf-1*	RING-finger	OBS	x	x
C11H1.3	.	RING-finger	Ahringer library	x	x
C12C8.3	*lin-41*	RING-finger	Ahringer library	x	
C15F1.5	.	RING-finger	OBS	x	
C16A3.7	*tag-182*	RING-finger	Ahringer library	x	
C16C10.5	*rnf-121*	RING-finger	Ahringer library	x	
C16C10.7	*rnf-5*	RING-finger	Ahringer & OBS	x	
C17E4.3	*marc-3*	RING-finger	Ahringer & OBS	x	
C17G1.4	*nra-3*	RING-finger	OBS	x	
C17H11.6	.	RING-finger	Ahringer library	x	
C18B12.4	.	RING-finger	Ahringer library	x	
C28H8.9	*dpff-1*	RING-finger	Ahringer library	x	
C32D5.10	.	RING-finger	Ahringer & OBS	x	
C32D5.11	.	RING-finger	Ahringer library	x	
C32E8.11	*ubr-1*	RING-finger	Ahringer library	x	
C34D4.14	*hecd-1*	HECT	Ahringer library	x	
C34E10.4	*wrs-2*	RING-finger	Ahringer & OBS	x	
C36A4.8	*brc-1*	RING-finger	Ahringer library	x	x
C49H3.5	*ntl-4*	RING-finger	Ahringer & OBS	x	x
C52E12.1	.	RING-finger	Ahringer library	x	
C53A5.6	.	RING-finger	Ahringer & OBS	x	
C56A3.4	.	RING-finger	Ahringer library	x	
D1081.9	.	RING-finger	OBS	x	
D2030.7	.	RING-finger	Ahringer & OBS	x	
D2085.4	.	HECT	Ahringer library	x	
D2089.2	*marc-2*	RING-finger	Ahringer & OBS	x	
F01F1.4	*rabn-5*	RING-finger	Ahringer & OBS	x	
F10D7.5	.	RING-finger	Ahringer library	x	x

F10G7.10	.	RING-finger	Ahringer library	x	
F11A10.3	*mig-32*	RING-finger	Ahringer library	x	
F14D12.2	*unc-97*	RING-finger	Ahringer & OBS	x	
F16A11.1	.	RING-finger	Ahringer & OBS	x	
F19G12.1	.	RING-finger	Ahringer library	x	
F20H11.1	.	RING-finger	Ahringer & OBS	x	
F26E4.11	*hrdl-1*	RING-finger	Ahringer library	x	
F26F12.7	*let-418*	RING-finger	Ahringer library	x	
F26G5.9	*tam-1*	RING-finger	OBS	x	x
F32A6.3	*vps-41*	RING-finger	Ahringer library	x	
F35G12.9	*apc-11*	RING-finger	OBS	x	
F36A2.13	*ubr-5*	HECT	Ahringer library and this study	x	
F36F2.3	*tag-214*	RING-finger	Ahringer library	x	
F36F2.3	*tag-214*	U-Box	Ahringer library	x	
F53F8.3	.	RING-finger	OBS	x	
F53G2.7	*mnat-1*	RING-finger	OBS	x	
F54B11.5	.	RING-finger	OBS	x	
F55A11.7	.	RING-finger	OBS	x	
F55A12.10	.	RING-finger	OBS	x	
F55A3.1	*marc-6*	RING-finger	Ahringer library	x	
F55G1.6	.	RING-finger	Ahringer & OBS	x	
F56A3.2	*slx-1*	RING-finger	Ahringer & OBS	x	
F56D2.2	.	RING-finger	Ahringer & OBS	x	
F58B6.3	*par-2*	RING-finger	Ahringer library	x	
F59E10.2	*cyp-4*	U-Box	Ahringer library	x	x
H05L14.2	.	RING-finger	Ahringer library	x	
K01G5.1	*tag-331*	RING-finger	Ahringer library	x	
K02B12.8	*zhp-3*	RING-finger	Ahringer library	x	
K04C2.4	*brd-1*	RING-finger	OBS	x	x
K08E3.7	*pdr-1*	RING-finger	Ahringer & OBS	x	
M02A10.3	*sli-1*	RING-finger	Ahringer & OBS	x	x
M110.3	.	RING-finger	Ahringer & OBS	x	
M142.6	*rle-1*	RING-finger	Ahringer library	x	x
R05D3.4	*rfp-1*	RING-finger	this study	x	
R05G6.4	.	U-Box	Ahringer & OBS	x	
R06F6.2	*vps-11*	RING-finger	Ahringer library	x	
R10A10.2	*rbx-2*	RING-finger	Ahringer & OBS	x	
T05A12.4	.	RING-finger	Ahringer & OBS	x	
T05H10.5	*ufd-2*	U-Box	Ahringer & OBS	x	
T08D2.4	.	RING-finger	Ahringer & OBS	x	
T09B4.10	*chn-1*	U-Box	Ahringer library	x	
T10F2.4	*prp-19*	U-Box	Ahringer library	x	x
T12E12.1	.	RING-finger	Ahringer & OBS	x	
T13A10.2	.	RING-finger	Ahringer & OBS	x	
T13H2.5	*spat-3*	RING-finger	this study	x	x
T14G8.1	*chd-3*	RING-finger	Ahringer & OBS	x	
T20F5.6	.	RING-finger	Ahringer library	x	
T20F5.7	.	RING-finger	Ahringer & OBS	x	
T24D1.2	.	RING-finger	Ahringer library	x	
T24D1.3	.	RING-finger	Ahringer library	x	
W02A11.3	*toe-4*	RING-finger	Ahringer & OBS	x	
W04H10.3	*nhl-3*	RING-finger	Ahringer library	x	
W06B4.3	*vps-18*	RING-finger	Ahringer library	x	
W09G3.6	.	RING-finger	Ahringer library	x	
Y105E8A.14	.	RING-finger	Ahringer & OBS	x	
Y2H9A.1	*mes-4*	RING-finger	Ahringer library	x	
Y37E11AR.2	*siah-1*	RING-finger	Ahringer & OBS	x	
Y38H8A.2	.	RING-finger	OBS	x	
Y39A1C.2	*oxi-1*	HECT	Ahringer library	x	
Y45F10B.8	.	RING-finger	Ahringer & OBS	x	
Y45F10B.9	.	RING-finger	Ahringer & OBS	x	
Y45G12B.2	.	RING-finger	Ahringer & OBS	x	
Y47D3A.22	*mib-1*	RING-finger	Ahringer library	x	
Y47G6A.14	.	RING-finger	Ahringer library	x	
Y48G8AL.1	*herc-1*	HECT	Ahringer library	x	

Y49F6B.9	.	RING-finger	Ahringer & OBS	x	
Y4C6A.3	.	RING-finger	Ahringer library	x	
Y51F10.2	.	RING-finger	this study	x	
Y52E8A.2	.	RING-finger	OBS	x	
Y53G8AR.5	.	RING-finger	Ahringer & OBS	x	
Y54E10BR.3	.	RING-finger	Ahringer library	x	
Y55F3AM.6	.	RING-finger	Ahringer & OBS	x	
Y57A10A.31	.	RING-finger	Ahringer & OBS	x	
Y65B4BR.4	*wwp-1*	HECT	OBS	x	x
Y67D8C.5	*eel-1*	HECT	OBS	x	
Y71F9AL.10	.	RING-finger	OBS	x	
ZC13.1	.	RING-finger	Ahringer & OBS	x	
ZK287.5	*rbx-1*	RING-finger	Ahringer & OBS	x	x
ZK637.14	.	RING-finger	Ahringer & OBS	x	

Table S1. List of RNAi clones used in this study

Biology Open • Supplementary information

organism					
S. cerevisiae	***H. sapiens I***	***H. sapiens II***	***H. sapiens III***	***T. brucei***	***C. elegans***
**associated function**					
splicing<br>transcription<br>DNA repair	splicing	splicing	splicing<br>transcription<br>DNA repair	splicing	??
**components**					
Prp19/Pso4	*PRP19/PSO4/SNEV*		*PRP19/PSO4/SNEV*	*PRP19*	*prp-19* *
Syf1/Ntc90		*XAB2/SYF1/HCRN*	*XAB2/HCRN*		*C50F2.3* *
Isy1/Ntc30		*ISY1/FSAP33*	*ISY1/FSAP33*		*F53B7.3* *
Cef1/Ntc85	*CDC5L*			*CDC5*	*cdc-5L* *
Prp46/Ntc50	*PRL1/PRP46/CWC1*			*PRL1*	*cpf-1* *
Snt309/Ntc25	*SPF27/SNT309/DAM1*			*SPF27*	*T12A2.7* *
Cwc15	*AD002/CWF15*				*T10C6.5*
Clf1/Ntc77/Syf3		*SYF3/CRNKL1/CLF*			*M03F8.3* *
Syf2/Ntc31		*SYF2/fSAP29/p29*			*K04G7.11* *
Cwc2/Ntc40		*RBM22/fSAP47/CWC2*			*T11G6.8*
Ntc20					
Yju2					*ZK1307.9*
				PRP17	*prp-17*
		PPIE/CypE/Cyclophilin-33	*PPIE/Cyclophilin-33*		*Y17G9B.4*
		hAquarius/fSAP164	*hAquarius/fSAP164*		*emb-4*
			CCDC16/ZNF830		
	CTNNB1 (beta-catenin)				
	HSP73				*hsp-1* *
		PRP45/SKIP/SNW1		*SKIP*	*skp-1*
		CCDC12			*C07A9.2*
		PPIL1/CYPL1		*PPIL1*	*Y69A2AR.21*
		GCIP			*cyn-12*

Table S2. Putative C. elegans homologs of the Prp19C/NTC complex
Putative *C. elegans* members of the Prp19C/NTC complex were identified based on orthologs in *S. cerevisiae*, *H. sapiens* and *T. brucei*. The core Prp19C/NTC members that were RNAi-depleted in *glp-1(rrr27)* animals are marked with “*”.
